# Development of a rapid antigen-based lateral flow assay for tick-borne spotted fever rickettsioses

**DOI:** 10.1371/journal.pone.0312819

**Published:** 2025-01-17

**Authors:** Richard Willson, Yingxin Zhao, Kristen Brosamer, Yogita Pal, Lucas S. Blanton, Esteban Arroyave, Carsen Roach, David H. Walker, Katerina Kourentzi, Rong Fang

**Affiliations:** 1 William A. Brookshire Department of Chemical and Biomolecular Engineering, University of Houston, Houston, Texas, United States of America; 2 Department of Internal Medicine, University of Texas Medical Branch, Galveston, Texas, United States of America; 3 Department of Internal Medicine, Division of Infectious Diseases, University of Texas Medical Branch, Galveston, Texas, United States of America; 4 Department of Pathology, University of Texas Medical Branch, Galveston, Texas, United States of America; University of Kentucky College of Medicine, UNITED STATES OF AMERICA

## Abstract

Tick-borne spotted fever rickettsioses (SFRs) continue to cause severe illness and death in otherwise-healthy individuals due to lack of a timely and reliable diagnostic laboratory test. We recently identified a diagnostic biomarker for SFRs, the putative N-acetylmuramoyl-l-alanine amidase RC0497. Here, we developed a prototype laboratory test that targets RC0497 for diagnosis of SFRs. The concentrations of RC0497 in sera of *Rickettsia rickettsii*-infected guinea pigs and *R*. *conorii*-infected mice were determined by stable isotope dilution–parallel reaction monitoring mass spectrometry (SID-PRM-MS), ranging from 0.1 to 1.1 ng/ml. Using europium chelate nanoparticle reporters, we developed a lateral flow assay (LFA) and evaluated the test with a panel of serum samples of mock and experimentally infected animals. Interestingly, 21 of 22 (95.5%) serum samples from *R*. *rickettsii*-infected guinea pigs and *R*. *conorii*-infected mice yielded positive results with a ratio of test line / control line greater than the cutoff value determined for non-infected animals. All uninfected samples were in agreement with the intended results, suggesting that the initially assessed specificity of the test is 100%, among these samples. Mice infected with a lethal dose of *R*. *conorii* and treated with doxycycline on day 3 post-infection (p.i.), upon RC0497 detection by LFA, displayed significantly decreased rickettsial loads, comparable to the sublethal infection group on day 5 p.i.. A panel of human serum samples spiked with various concentrations of recombinant RC0497 were analyzed by LFA, suggesting that the limit of detection of the LFA was 0.64 ng/mL. These findings suggest that the timely detection of RC0497 by a europium LFA offers guidance for treatment, leading to a significant improvement in infection outcomes. This work, for the first time, shows significant promise for a rapid and easy-to-use platform offering a timely diagnostic assay for severe SFRs.

## Introduction

Rickettsiae are Gram-negative and obligately intracellular bacteria primarily targeting microvascular endothelial cells. Rickettsial diseases are a group of arthropod-associated infections caused by pathogenic *Rickettsia* species, mostly belonging to the spotted fever group (SFG) and typhus group (TG) [1]. Spotted fever rickettsioses (SFRs) are transmitted to humans by infected ticks, such as *Dermacentor variabilis*, *Amblyomma maculatum* and *Rhipicephalus sanguineus* [2]. According to the CDC, the incidence of SFRs has risen dramatically in the last two decades, possibly due to changes in climate and expansion of the tick population [3,4]. Although SFRs can occur at any time of the year, most cases are reported in months when ticks are most active, April through October. In the United States, SFRs occur in 47 states with the highest incidence observed in Arkansas, Missouri, Oklahoma, North Carolina, Tennessee, Virginia, and Arizona [5–11]. SFRs manifest with a diverse spectrum of symptoms ranging from very mild to life-threatening or even fatal infections. While *R*. *parkeri* rickettsiosis and African tick-bite fever are usually milder or even undiagnosed, Rocky Mountain spotted fever (RMSF) and Mediterranean spotted fever (MSF, also called boutonneuse fever) represent the most severe forms of SFRs [1]. MSF, caused by *R*. *conorii*, is an endemic disease prevalent in many countries with fatal cases occasionally reported, such as in Tunisia, Spain, Portugal, Italy, France, Israel, India, and Iran [12–25]. RMSF, caused by *Rickettsia rickettsii*, is the world’s deadliest tick-borne disease with untreated and treated case fatality rates of 23% and 4%, respectively [26]. Recent reports in Brazil and Mexico show that the case fatality rates of RMSF can be as high as 50% [27,28]. In addition to cases of RMSF and *R*. *parkeri* rickettsiosis, Pacific Coast tick fever caused by *Rickettsia* strain 364D has been reported in California, US [29–31]. Until now, no FDA-licensed vaccine against rickettsioses is available.

Despite the availability of antibiotics to which rickettsiae are sensitive, severe SFRs result in significant mortality and long-term sequelae, such as impaired hearing, neurocognitive deficits, cutaneous necrosis, and gangrene of extremities requiring amputation [1,32]. The clinical course of SFRs typically initiates following exposure to ticks or contact with dogs. Within an incubation period of approximately 3~12 days, patients with SFRs undergo a sudden onset of symptoms, including fever, headache, chills, malaise, and myalgia. Within two to three days following the onset of initial symptoms, patients may or may not develop a maculopapular rash, vomiting, abdominal pain, and cough. These non-specific symptoms can also be attributed to various viral, bacterial, or parasitic pathogens. Depending on the virulence of the rickettsial organisms and whether treatment is given in a timely manner, SFRs can progress to an overwhelming infection involving multi-organ injury or even failure such as pneumonitis, acute respiratory distress syndrome (ARDS), myocarditis, meningo-encephalitis, acute kidney injury, and death. It is important to note that half of RMSF-related deaths occur within 9 days of symptom onset [33–35]. Early diagnosis and treatment with doxycycline within 5 days of illness onset are crucial for preventing fatalities in RMSF and reducing complications in other SFR cases [33,36].

Rickettsial diseases are difficult to diagnose due to the lack of a reliable and timely laboratory test for confirmation. At present, detection of antibodies against rickettsiae in the serum by indirect immunofluorescence assay (IFA) is the gold standard for diagnosis of tick-borne rickettsioses [1]; however, the antibody response typically occurs 7 to 10 days after the onset of illness, when deaths begin to occur. Furthermore, detection of antibodies in a single serum specimen is not diagnostic, as antibodies are present in the sera of more than 10% of healthy individuals [37]. While molecular detection of bacterial DNA and immunohistochemical analysis of rickettsial antigen in the skin biopsy specimens could yield an early diagnosis, the proportion of cases with skin rash and the onset of rash are not reliable, and few hospital laboratories perform rapid-turnaround molecular diagnostic testing for SFRs [1]. Pathogenic rickettsiae primarily infect the microvascular endothelial cells and may not circulate in large numbers in the blood until the disease has progressed to a severe phase of infection [38]. Additionally, routine hospital blood cultures cannot effectively detect obligately intracellular rickettsiae. Laboratory isolation and culture of rickettsiae from clinical samples for diagnosis require technical expertise and specialized biosafety level-3 laboratory facilities. Thus, no convenient, specific, acute-phase laboratory diagnostic tests are available for SFRs, which makes these diseases not only notoriously difficult to diagnose but also severely underreported.

To this end, we recently identified a rickettsial protein, RC0497, in *R*. *conorii*-infected endothelial cell supernatant. RC0497 is a putative N-acetylmuramoyl-L-alanine amidase of *R*. *conorii*, which is highly conserved across different SFG species [39]. We further demonstrated that RC0497 is present in the circulating blood of an infected mouse model of MSF and patients with MSF [39]. In the present study, by using two experimental models of SFRs, we evaluated the hypothesis that detection of RC0497 by a rapid lateral flow assay (LFA) will provide a timely diagnostic assay which can potentially improve treatment outcomes for severe SFRs. Two animal models of SFRs, *R*. *conorii*-infected C3H/HeN mice and *R*. *rickettsii*-infected guinea pigs, mimic the pathophysiologic changes in patients with RMSF [40,41]. We first determined the concentrations of RC0497 at different time points of disease by mass spectrometry (MS) of blood specimens from infected animals. With specific antibodies against RC0497 and europium chelate nanoparticle reporters, we developed a europium nanoparticle LFA (EuNP LFA) to detect this diagnostic marker for SFRs. This assay performed with high analytical sensitivity and specificity in serum samples of experimentally infected animals at the early stage of infection, when rickettsiae could be controlled by antibiotic treatment.

## Materials and methods

### *Rickettsia rickettsii* and *Rickettsia conorii*

*Rickettsia conorii* (Malish 7 strain) and *R*. *rickettsii* (Sheila Smith strain) were purchased from the American Type Culture Collection (ATCC) followed by culture and storage at -80°C. The concentrations of rickettsial stocks were determined by plaque assay and/or quantitative real-time PCR as described previously [42,43]. For mouse inoculation, *R*. *conorii* were first cultivated in specific-pathogen-free embryonated chicken eggs followed by limited passage in Vero cells. *R*. *conorii* were harvested, purified, and resuspended in sucrose-phosphate-glutamate (SPG) buffer (0.218 M sucrose, 3.8 mM KH_2_PO_4_, 7.2 mM K_2_HPO_4_, 4.9 mM monosodium glutamic acid; pH 7.0) as described previously [42–45]. The virulence of *R*. *conorii* stock was evaluated by illness and infection outcomes after inoculating mice intravenously [46]. *Rickettsia rickettsii* Sheila Smith strain was cultivated in Vero cells, and guinea pigs were inoculated intraperitoneally with the cell-cultured *R*. *rickettsii*. When guinea pigs displayed signs of illnesses (fever, weight loss, and scrotal erythema), spleen and blood were collected, homogenized, and stored as a 50% *R*. *rickettsii*-infected guinea pig spleen-blood suspension in SPG [47]. This stock was stored at -80°C until use.

### Experimental models of spotted fever rickettsioses

Wild-type (WT) C3H/HeN mice (6- to 8-week-old) and Hartley strain guinea pigs (500 grams) were purchased from Charles River Laboratories Inc. (Wilmington, MA, USA). All animals were housed in an animal biosafety level 3 laboratory facility at the University of Texas Medical Branch, Galveston. All experiments and procedures were approved by the University of Texas Medical Branch Animal Care and Use Committee, and animals were used according to the guidelines in the Guide for the Care and Use of Laboratory Animals. All the methods for animals have been selected based on their effectiveness and adherence to ethical standards for animal welfare. C3H/HeN mice were infected intravenously as described previously [43–45] using a lethal dose (LD) of 1.5 × 10^5^ PFU (3 × 50% lethal doses, 3 LD_50_) or a sublethal dose (SLD) of 1.5 × 10^4^ PFU (0.3 × 50% lethal dose, 0.3 LD_50_) of *R*. *conorii*. Guinea pigs were inoculated intraperitoneally with a previously determined lethal dose of *R*. *rickettsii* (3 x 10^3^ infectious organisms) prepared by mixing sterile PBS with the stock described above [47]. Mock-infected animals were inoculated with the same volume of PBS buffer in the same manner as controls. Each group included 3~5 animals.

Upon inoculation with rickettsiae, animals were monitored daily for weight loss and other sings of illness such as decreased mobility, roughened fur, and lethargy [46]. If signs of illness were present, we assessed the illness score as previously described [46] and increased the monitoring frequency to two to three times a day, with 6–8 hours between observations. Each team member received comprehensive training and instructions to conduct all animal checks during the light cycle. Sick animals had food placed on the cage floor. Intervention was avoided to allow the disease to progress naturally for full characterization and experimental evaluation. Animals were humanely euthanized at the indicated time points for collection of serum and/or tissue samples. For serum collection, blood was drawn into standard 1.5-ml Eppendorf tubes (Thermo Fisher Scientific, US). After incubating at room temperature for 1 hour, the samples were centrifuged at 3,000 rpm (1,500 × g) for 15 minutes at 4°C. The supernatant was collected as serum samples. Depending on the inoculum, infected animals were either euthanized or recovered after 15 days. The maximal duration of the experiments was one month. Mice exhibiting greater than 20% weight loss, accompanied by signs of distress such as moribund behavior or neurological signs (such as seizures, tremors, head tilt, or paralysis), were euthanized promptly. Guinea pigs displaying severe lethargy, characterized by a lack of response to stimulation, an inability to access food or water sources, or experiencing weight loss exceeding 20%, were also promptly euthanized. Humane euthanasia methods included 5% CO_2_ followed by cervical dislocation for mice and bilateral thoracotomy for guinea pigs.

### Generation of specific antibodies against RC0497

Recombinant protein RC0497 was expressed in 6x Histidine-tagged form in *Escherichia coli* (*E*. *coli*) transformed by the constructed pET-28a (+) vector [39], which was provided as a gift by Dr. Sanjeev Sahni at the University of Texas Medical Branch at Galveston. The expression and purification of protein RC0497 were performed in house or by Genscript Biotech Corp. (Piscataway, NJ). Briefly, after expression and removal of cell debris in the cell lysates, the protein was purified using Ni-NTA chromatography and dialyzed to remove imidazole and benzamidine to optimize thrombin activity, followed by removal of the His-tag with thrombin. Rabbit polyclonal antibodies against the recombinant RC0497 were generated by Rockland Immunochemicals, Inc. (Limerick, PA). The titers of polyclonal antibodies against RC0497 were determined before collection of blood from immunized rabbits. The antibodies were further purified by protein A affinity chromatography. The reactivity of the polyclonal antibodies against RC0497 was confirmed by immunoblotting and ELISA.

### Immunoprecipitation (IP) of RC0497 and trypsin digestion

Immunoprecipitation (IP) of RC0497 was performed as described previously [39]. Briefly, about 100 μL of uninfected and infected serum was suspended in 1 mL of low-ionic-strength IP buffer (50 mM NaCl, 25 mM HEPES pH 7.4, 1% IGEPAL CA-630, 10% glycerol, 1 mM fresh dithiothreitol (DTT), and protease inhibitor cocktail). An aliquot of 4 μg of anti-RC0497 antibody or control IgG (rabbit polyclonal, Santa Cruz) was used in each IP. The mixture was incubated overnight at 4°C, and then 30 μL of protein A magnetic beads (Dynabeads, Invitrogen) were added. After incubation at 4°C for 4 h, the beads were separated from the supernatant with a magnetic stand. The beads were washed with PBS five times before trypsin digestion. The trypsin digestion was performed as previously described [48].

### Quantification of RC0497 by stable isotope dilution–parallel reaction monitoring mass spectrometry (SID-PRM-MS)

An aliquot of stable isotope standard mass-labeled peptide (SIS peptide) of *R*. *conorii* protein RC0497 (LLLSLDSTGEK [^13^C_6_^15^N_2_]) was added to each sample. For PRM analyses, the peptides were analyzed with an Easy nLC1000 UHPLC-Q Exactive Orbitrap LC-MS system (Thermo Scientific, San Jose, CA). The full scan resolution was 70,000 (@m/z 200), the target AGC value was set to 3 x 10^6^, and maximum fill time was 200 ms for full scan; 17,500 (@m/z 200), a target AGC value of 2 x 10^5^, and maximum fill times of 100 ms for MS2 scan. PRM targeted the paired native and SIS peptides of *R*. *conorii* protein RC0497. Assessment of peptide detection was performed post-acquisition using Skyline version 3.6.0.9321 [48,49]. For each peptide evaluated, the signals of the five most intense fragment ions (as defined in spectra of SIS peptides of RC0497) were extracted from each corresponding MS/MS spectrum. The MS/MS spectra with five fragment ions detected were submitted to spectral matching. The comparison of the relative intensities of these fragments with those defined in the reference composite MS/MS spectrum was performed based on the dot product value. In addition, the retention time of the native and SIS peptides was used as an additional acceptance criterion. The variation of the retention time between the analyte peptides and their SIS counterparts should be within 0.05 min.

### Rickettsial concentrations in mouse tissues determined by real-time PCR

To demonstrate whether detection of RC0497 can guide timely and effective antibiotic treatment, we utilized quantitative real-time PCR to determine the concentration of rickettsiae in liver and lung of mice infected with a lethal dose of *R*. *conorii* and treated with doxycycline at the time when RC0497 was detected. Tissue samples of uninfected mice served as controls. First, genomic DNA was extracted using a Qiagen DNA extraction kit (Valencia, CA, USA) as described previously [43,44]. Quantitative real-time PCR was performed with primers and TaqMan probes for the *Rickettsia*-specific citrate synthase (CS) gene (*gltA*) as described in our previous studies: *gltA* forward, GAGAGAAAATTATATCCAAATGTTGAT; *gltA* reverse, AGGGTCTTCGTGCATTTCTT; *gltA* probe, CATTGTGCCATCCAGCCTACGGT. The *gltA* probe was labeled with 6-carboxyfluorescein (FAM). Quantitative real-time PCR was performed using an iCycler (Bio-Rad, Hercules, CA, USA) as described previously [42–46]. Two-step cycle parameters (95°C and 60°C) were used. The results were normalized to the amount (in nanograms) of genomic DNA in the same sample and expressed as CS gene copy number per nanogram of genomic DNA.

### Antibiotic treatment and quantification of bacterial load in tissues

C3H/HeN mice infected with a lethal dose of *R*. *conorii* were treated with doxycycline (Mylan Institutional Inc., NDC#67457-437-00) at a dosage of 40 mg/kg body weight two times a day intraperitoneally starting on day 3 p.i. when RC0497 was detected as described previously [50,51]. On day 5 p.i., mice were euthanized, and tissues, including liver and lung, were collected for determination of bacterial concentrations. Lethally and sub-lethally infected mice treated with vehicles such as PBS served as controls.

### Nanoparticle LFA reporter functionalization

Carboxylate-modified polystyrene particles containing encapsulated europium chelate (Excitation 365 nm/ Emission 610 nm; Thermo Fisher, 200 nm FluoSpheres #F20881 [used initially] or Bangs Laboratories, 200 nm, #FCU002 [used in the “Version 2" LFA, see below]) were functionalized with rabbit polyclonal antibodies against RC0497 using standard EDC-NHS (1-ethyl-3-[3-dimethylaminopropyl] carbodiimide and N-hydroxysuccinimide) chemical activation.

Briefly, particles at 0.5% solids were centrifuged (10 min at 16,500 × g) and washed twice with 50 mM 2-N-morpholino ethanesulfonic acid (MES) buffer, pH 5.8. The particles were resuspended in 95 μL of MES buffer and sonicated until no visible aggregates remained. Particles were activated by EDC (1-ethyl-3-(3-dimethylaminopropyl) carbodiimide hydrochloride, Thermo Fisher; #A35391) and NHS (Millipore Sigma; #130672) at a molar ratio of 20 NHS per carboxyl group (3.4 μL of 50 mg/mL NHS) and 2.5 EDC per carboxyl group (3.5 μL of 10 mg/mL EDC). NHS and EDC were added in that order to the resuspended particle mixture which was then placed on a benchtop rotator for 30 min at 20°C. After activation, the particles were washed twice by centrifugation in 1× PBS and resuspended by sonication. Twenty-five μg of antibody (25 μL of 1 mg/mL suspended in 1× PBS) were added to the particles and incubated at 20°C on a benchtop rotator for 2 h. Then, the mixture of particles and antibodies was centrifuged (10 min at 16,500 × g), the supernatant was removed, and particles were resuspended in 4% bovine serum albumin (BSA) in 1 × phosphate buffered saline (PBS) and incubated overnight at 20°C on a benchtop rotator. The particles were washed three times by centrifugation with 1× PBS, 1% BSA solution, resuspended at 0.5% solids in 100 μL 1× PBS, 1% BSA, and stored at 4°C.

### Lateral flow strip preparation

FF120HP nitrocellulose membrane (Cytiva; #13549205) was cut to a width of 2.5 cm using a craft paper cutter and assembled onto a 30-mm long, 60-mm wide backing adhesive (DCN Diagnostics; #MIBA-020 or MDI; #L-P25) along with a CF5 absorbent pad (Cytiva; #8115–2250) and a sample pad (Ahlstrom; ReliaFlow, #8980). The assembled membrane was striped using a BioDot dispenser (BioDot; #XYZ30600124), with a flow rate of 1 μL/cm and a dispense volume of 30 μL per 30-cm card. The test line (TL) contained rabbit polyclonal antibodies against RC0497 striped at 1 mg/mL in 1× PBS/ 0.2% sucrose, and the control line (CL) contained goat anti-rabbit antibodies (Arista; #ABGAR-0500) striped at 1 mg/mL in 1× PBS/ 0.2% sucrose. Striped membranes were dried in a Robbins Scientific Micro Hybridization Incubator 2000 at 37°C for 30 min, then dried overnight at 20°C in a desiccator chamber (Totech; SuperDry Desiccant Cabinet; #SD-151-21) at 5% relative humidity. The striped card was then cut into 3-mm strips using a KinBio ZQ2000 Guillotine Cutter and stored at 20°C in sealed 50-mL conical tubes (USA Scientific; #5622–7261) with desiccant packs (Interteck Packaging; #IN1G51).

### Lateral flow assay

The LFA running buffer for dilution of serum samples (“LFA buffer”) contained 1× PBS (pH 7.4), 1% BSA (Millipore Sigma; #A9418), 1% IGEPAL CA-630 (Millipore Sigma; #I8896), 1% casein (Millipore Sigma; #C5679) and 0.3% PEG 3000 (Millipore Sigma; #81227). The LFA buffer was made at least 1 day before use and left to rotate at room temperature overnight to allow casein to dissolve completely. For confirmation of RC0497 detection, purified recombinant RC0497 protein (Genescript; #LOC107446314) was spiked in 60 μL running buffer or running buffer/human serum mixture (serum from healthy donors was obtained from Gulf Coast Regional Blood Center, Houston, Texas, and stored at −20 °C until used), mixed with europium particle reporters (EuNPs; 1.5 μL, 0.5% solids), and pipetted onto the sample pad in two steps (30 μL each). After the sample wicked through, strips were washed with 15 μL LFA buffer before imaging (after 15 min).

Serum samples from infected animals (15 μL each) were diluted to 25% in LFA buffer and mixed with 1.5 μL EuNPs (0.5% solids). 60 μL of sample was pipetted onto the sample pad in two steps (30 μL each) then washed with 15 μL of LFA buffer before imaging after 15 minutes.

To determine the limit of detection (LoD) of RC0497-LFA in human serum samples, purified recombinant RC0497 was diluted in human serum samples from healthy donors at a range of concentrations including 0, 0.05, 0.1, 0.2, 0.5, 1, 2, 5 and 10 ng/mL. We included five replicates for each concentration of RC0497 in human sera. Strips were run, imaged, and analyzed as described above and below.

### LFA strip imaging and analysis

During LFA development, in addition to analysis by LFA reader, strips were imaged by a CoolSNAP K4 CCD 2,048 × 2,048-pixel camera (Photometrics, Blaine WA) controlled by Micro-Manager 1.4.22 software (Vale Lab, University of California, San Francisco CA) in a FluorChem SP gel cabinet (Alpha Innotech Corp., San Leandro CA). The strips were optically excited with the built-in epi-illuminating UV light and imaged through an originally-equipped UV cut-off filter with an exposure time of 1 sec and pixel binning of 4.

LFA strips were then analyzed using a portable Qiagen ESEQuant LR3 fluorescence LFA reader (equipped with two europium detection channels of differing sensitivity) or a Lumigenex time-resolved fluorescence LFA analyzer (LTRIC-600). Intensity profiles along the length of the LFA strips were recorded and analyzed on a PC using each reader’s proprietary software, Lateral Flow Studio (Qiagen, version 3.6.2) or LReader (Lumigenex, version 8.5). The calculated integrated area under the curve for each peak (TL and CL) was transferred to MS Excel, and average TL/CL ratios with associated standard deviation values were calculated.

### Statistical analysis

For comparison of multiple experimental groups, one-way analysis of variance (ANOVA) with Bonferroni’s procedure was used and analyzed statistically with GraphPad Prism software version 9.1.1 (GraphPad Software, San Diego, CA, USA). To compare two groups, the unpaired t-test was used. *P* values of 0.05 or less were considered significant. Survival differences were compared using Kaplan-Meier survival curves, followed by a log rank test.

## Results

### Development of a europium LFA for detection of diagnostic biomarker RC0497 in serum

**Fig 1:**
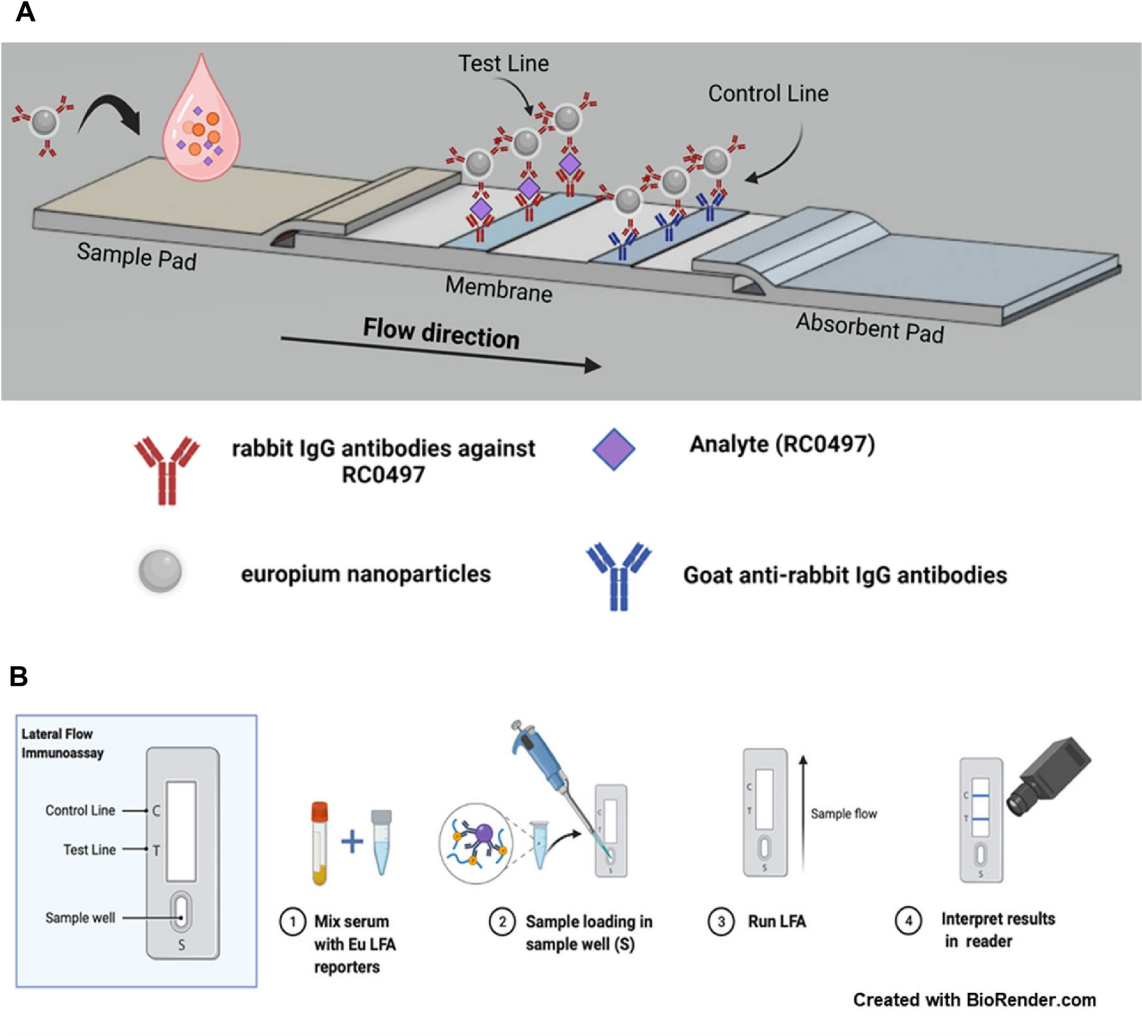
Schematic diagram of a europium nanoparticle lateral flow assay for detection of rickettsial protein RC0497. (A) The strip was composed of a nitrocellulose membrane assembled on an adhesive backing card along with a sample pad and absorbent pad. The assembled strip was printed with a test line (containing rabbit polyclonal anti-RC0497 IgG antibodies) and a control line (containing goat polyclonal anti-rabbit IgG antibodies). (B) Sample was first mixed with anti-RC0497 europium LFA particle reporters followed by loading on the sample pad. Wash buffer was then added and the LFA strip was read 15 min later in a europium LFA reader. Two different point-of-care LFA readers were evaluated.

We developed a europium nanoparticle reporter (EuNP) lateral flow assay for the detection of RC0497 in serum samples (Fig 1). A small volume of serum sample (15 μL) was diluted (1:4 in LFA buffer) followed by mixing with EuNPs (1.5 μL, 0.5% solids) functionalized with anti-RC0497 rabbit polyclonal IgG antibodies. When loaded onto the sample pad, the sample (containing any RC0497 analyte present captured on the EuNPs) migrated along the nitrocellulose membrane and was captured by the anti-RC0497 antibodies on the test line (TL) while rabbit IgG-coated EuNPs were captured on the control line (CL) bearing goat anti-rabbit IgG antibodies. The LFA strips were washed and imaged, and fluorescent signals on the test and control lines analyzed by the LFA fluorescence reader(s).

### Quantification of RC0497 by SID-PRM-MS and detection of RC0497 by EuNPs LFA in a guinea pig model of Rocky Mountain spotted fever (RMSF)

**Fig 2:**
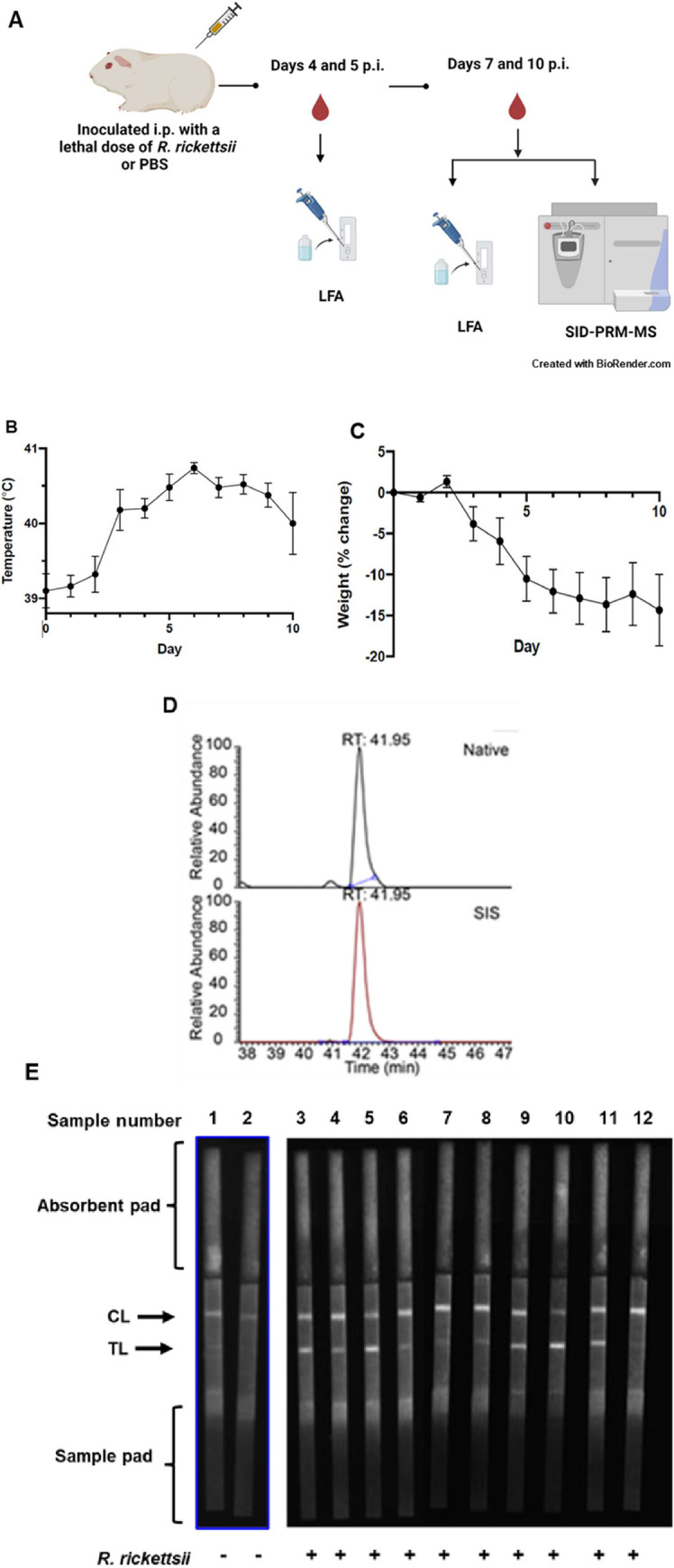
Detection of RC0497 by SID-PRM-MS and europium nanoparticle lateral flow assay in guinea pig model of spotted fever rickettsioses. Guinea pigs were inoculated intraperitoneally (i.p.) with *R*. *rickettsii* at a lethal dose of 3 x 10^3^ PFU (n = 5) (A). Animals inoculated with PBS served as controls. After infection, serum was collected on days 4, 5, 7 and 10 post infection (p.i.) for analysis by stable isotope dilution-parallel reaction monitoring mass spectrometry (SID-PRM-MS) and/or lateral flow assay (LFA), respectively. Upon infection, animals showed fever (B) and weight loss (C). Quantification of RC0497 was performed by SID-PRM-MS. (D) The upper panel is the extract ion chromatogram of native RC0497 peptide LLLSLDSTGEK; the lower panel is the extract ion chromatogram of stable isotope labeled LLLSLDSTGEK. (E) Detection of RC0497 in serum of infected and uninfected guinea pigs by LFA. FluorChem-based images of the strips that were later analyzed in a Qiagen ESEquant LR3 europium LFA reader.

To evaluate the performance of EuNP LFA, we first employed a guinea pig experimental model of RMSF (Fig 2A). Compared to mock infected controls, guinea pigs inoculated intraperitoneally with *R*. *rickettsii* developed fever (greater than 40°C) and significant weight loss (≥5%) on day 3~4 p.i. (Fig 2B and 2C). Other signs of illness included scrotal reaction (swelling, erythema, and eventually petechiae), lethargy, conjunctivitis, and ruffled fur. The illness of these animals became progressively worse over time until the guinea pigs were humanely euthanized between days 7 and 10 post infection (p.i.). Sera were collected on days 7 and 10 p.i. and appropriately stored for quantification of RC0497 by SID-PRM-MS assay (Fig 2A). Serum samples collected on days 4, 5, 7 and 10 p.i. were stored for detection of RC0497 by EuNP LFA (Fig 2A).

For quantification of RC0497 in sera of the experimental model of RMSF, two stable isotope-labeled RC0497 signature peptides as internal standards (SIS), LLLSLDSTGEK and SDFPAEQIGK, were custom-synthesized using isotopically labeled lysine, [^13^C_6_^15^N_2_] Lys (99% isotopic enrichment). The peptides were HPLC-purified and were stringently tested to ensure high purity (>97%). The accurate molecular weights of SIS peptides were measured via mass spectrometry, and the specific peptide content was determined by amino acid analysis. The concentration precision of these peptides was ±10~25%. These stable isotope-labeled peptides were spiked into a series of dilutions of unlabeled RC0497 peptides and used as an internal standard to determine the concentration of RC0497 in the specimens. The calibrants were analyzed with a QExactive Orbitrap mass spectrometer. Linear regression analysis of the observed peak area ratios (native:SIS) versus RC0497 concentration (Fig 2D) was performed to generate the calibration curve (coefficient of determination R2 > 0.99). The concentration of RC0497 in guinea pig serum was found to be about 0.12 ng/mL (n = 2) on days 7 and 10 p.i. with no significant difference in RC0497 concentration at these two time points.

Detection of RC0497 by EuNP LFA was first evaluated with a panel of 12 guinea pig serum samples, including two uninfected and ten *R*. *rickettsii*-infected specimens. All serum samples resulted in a fluorescent signal on the control line (Fig 2E), suggesting that the samples migrated along the membrane and the assay functioned correctly. The reader-determined TL/CL values were indistinguishable from 0 for the two uninfected guinea pig serum samples. The TL/CL values for all the infected guinea pig serum samples, including those not presented in Fig 2, were readily distinguishable from those of the negative samples (range: 0.08–1.20, median: 0.30, mean: 0.44, SD: 0.39) (S1 Table).

### Detection of RC0497 by SID-PRM-MS and europium LFA at the early stage of infection in murine model of spotted fever rickettsiosis (SFR)

**Fig 3:**
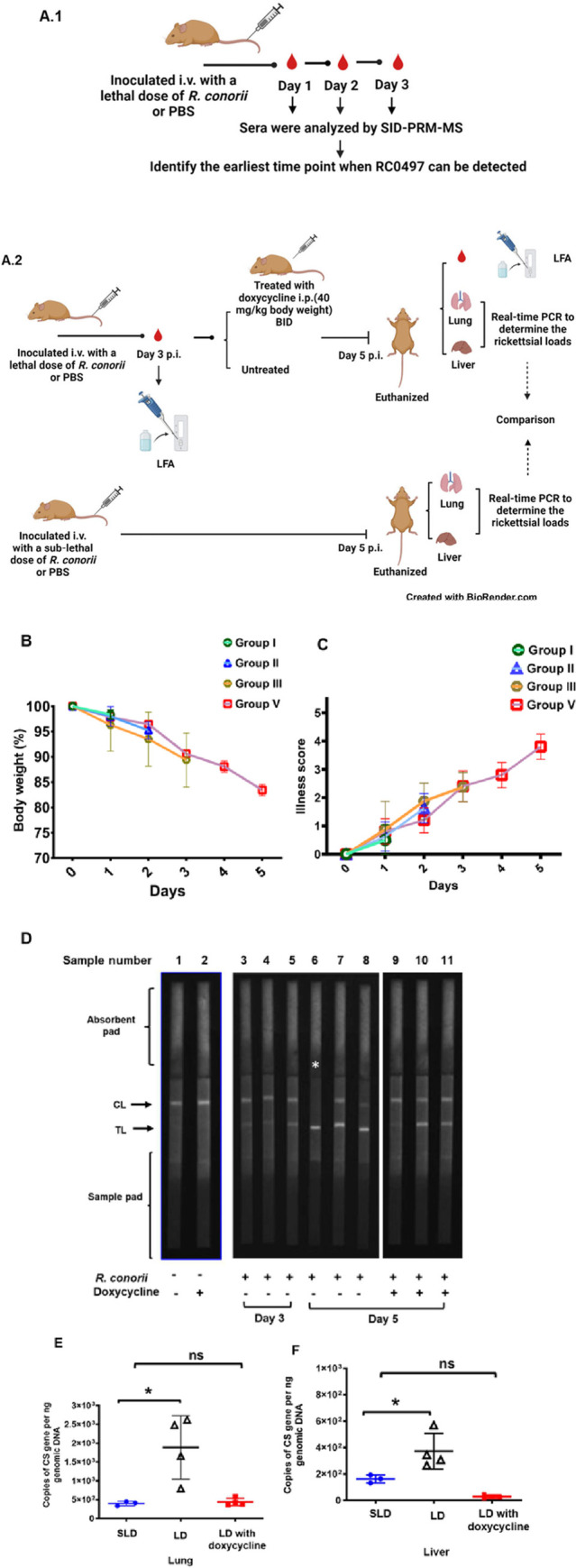
Determine the earliest time point when RC0497 was detected by europium nanoparticle LFA in murine spotted fever rickettsioses and its significance in guiding anti-rickettsial treatment. (A) C3H/HeN mice were intravenously (i.v.) inoculated with *R*. *conorii* and monitored daily (B and C). PBS-inoculated mice served as controls. (A.1) Mice were inoculated with a lethal dose (LD) of 1.5 × 10^5^ PFU per mouse. On days 1, 2 and 3 post infection (p.i.), groups of animals (Groups I, II and III, as shown in B and C) were euthanized individually for collection of serum. The collected serum was then analyzed by SID-PRM-MS to identify the earliest time point at which RC0497 can be detected. (A.2) Another panel of LD-infected mice were treated with doxycycline on day 3 p.i., as indicated in the Materials and Methods section, after blood collection. On day 5 p.i., these LD-infected mice (Group V in B and C) were euthanized. Sera collected on both days 3 and 5 p.i. were analyzed by LFA for detection of RC0497. Mouse tissues such as lung and liver were collected to determine the concentrations of *R*. *conorii* by quantitative real-time PCR. In parallel, a group of mice inoculated with a sub-lethal dose (SLD) of 1.5 x 10^4^ PFU per mouse were euthanized on day 5 p.i.. Mouse tissues were collected for evaluation of rickettsial loads. (D) FluorChem-based images of europium LFA strips for the detection of RC0497 in sera of LD-infected and uninfected mice. The LFA strip marked with a white asterisk (*) failed to show a detectable CL. (E and F) On day 5 p.i., concentrations of *R*. *conorii* in lung and liver of LD-infected, LD-infected and doxycycline-treated, and SLD-infected mice were determined by quantitative real-time PCR amplifying citrate synthase (CS) gene. Each group included 3 to 5 mice. ns, not statistically significant. *, *p*<0.05.

To determine how early RC0497 can be detected in the serum over the course of SFR, we employed a mouse model of severe SFR by intravenously inoculating C3H/HeN mice with *R*. *conorii* (Fig 3A) [40]. Mice i.v. inoculated with a lethal dose (LD) of *R*. *conorii* were euthanized on days 1, 2 and 3 p.i. for collection of serum, which was analyzed by SID-PRM-MS, individually, (Fig 3A.1). In parallel, a panel of C3H/HeN mice i.v. inoculated with LD of *R*. *conorii* were treated with doxycycline on day 3 p.i. and euthanized on day 5 p.i. to determine whether detection of RC0497 after 3 days of infection is timely enough for antibiotic treatment (Fig 3A.2). Mice inoculated with a lethal dose of *R*. *conorii* started to show weight loss and illness on day 3 p.i. (Fig 3B and 3C). Mice underwent continued weight loss and increasing illness score, resulting in death on days 6–7 post infection, consistent with our previous studies [44,46]. We did not detect any signal of RC0497 on days 1 and 2 p.i. by SID-PRM-MS. Interestingly, on day 3 p.i., RC0497 was detected in serum of mice inoculated with LD of *R*. *conorii* at a concentration of about 1.0 ng/mL (n = 4). In line with the results obtained by SID-PRM-MS, all serum samples collected from LD-infected mice on day 3 p.i. also tested positive for RC0497 by the EuNP LFA with a TL/CL greater than 0 (Fig 3D) (S1 Table).

### Assessment of whether detection of RC0497 by EuNP LFA is clinically actionable to guide treatment with antibiotic

Current laboratory diagnosis mostly relies on serology, which becomes positive on or after day 7 of illness when deaths have already begun to occur. As shown in Fig 3A.2, we infected WT C3H/HeN mice i.v. with a lethal dose of *R*. *conorii* and subsequently treated them with doxycycline on day 3 p.i., when RC0497 had appeared in the serum and could be detected by both SID-PRM-MS and LFA (Fig 3D). Among six serum samples collected from *R*. *conorii*-infected mice on day 5 p.i., all showed positive LFA signals (Fig 3D), in infected mice with or without doxycycline treatment. In contrast, the serum samples from uninfected mice with or without doxycycline treatment gave negative LFA results (Fig 3D). One infected mouse serum sample gave a fluorescence signal in TL, but not in CL. This sample was excluded from further analysis.

In parallel, we intravenously inoculated a group of mice with a sublethal dose of *R*. *conorii* (Fig 3A.2). These mice developed much milder illness and were humanely euthanized on day 5 p.i.. As shown in Fig 3E and 3F, compared to sublethal infection, the concentrations of *R*. *conorii* in both lung and liver were significantly greater in lethally infected mice. On day 5 p.i., rickettsial loads in tissues of doxycycline-treated mice were significantly less than in untreated mice. Interestingly, the concentration of rickettsiae in treated mice inoculated with a lethal dose of *R*. *conorii* was comparable to those in the untreated sublethal mouse group.

### Evaluation of performance of RC0497 by EuNP LFA

We assessed the diagnostic performance of the RC0497-LFA using the samples collected from experimental models of SFRs as described previously [52]. Of all 29 samples tested, 7 samples were from uninfected animals while 22 samples were from experimentally infected animals, including those that were tested or retested but not presented (N.P.) in Figs 2 and 3 (S1 Table). Negative/uninfected samples resulted in 100% agreement between test result and expected result, yielding a specificity of 100% (Table 1). 21 of 22 positive samples were in agreement with expected results, suggesting a sensitivity of 95%. The positive predictive value was 100% while the negative predictive value was 87.5%. Overall, the RC0497-LFA had excellent accuracy including specificity and sensitivity, exceeding minimal criteria of 90%.

**Table 1:** Evaluation of analytical specificity and sensitivity of RC0497-europium LFA using samples of experimentally infected models of spotted fever rickettsioses.

Tested positive	21 (True positive)	0 (False positive)
Tested negative	1 (False negative)	7 (True negative)

Sensitivity = Number of true positive samples/(number of true positive + number of false negative)

= 21 / (21+1) × 100 = 95.5%

Specificity = Number of true negative samples/(number of true negative + number of false positive)

= 7 / (7+0) × 100 = 100%

Positive predictive value (PPV) = Number of true positive samples/total number of tested positive

= 21 / (21+0) × 100 = 100%

Negative predictive value (NPV) = Number of true negative samples/total number of tested negative

= 7 / (7+1) × 100 = 87.5%

**Fig 4:**
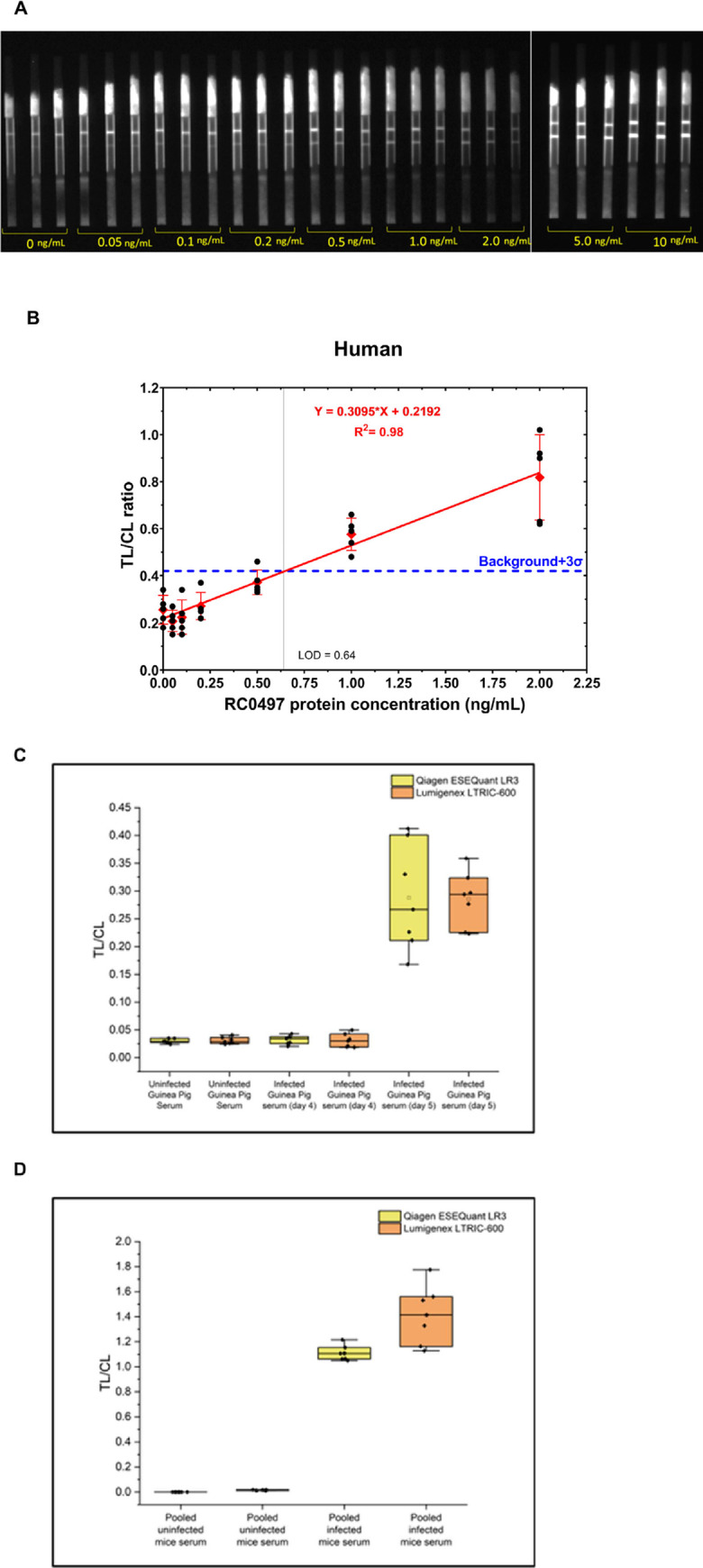
Evaluation of RC0497-LFA in human spiked samples and robustness of the RC0497 “version 2” LFA. A commercially-available recombinant RC0497 protein of high purity was spiked in human serum (diluted 25% in LFA running buffer) prior to analysis by LFA strips and the point-of-care, time-resolved europium Lumigenex LTRIC-600 reader. (A) Representative images of RC0497-LFA strips of human sera spiked with different concentrations of recombinant RC0497. (B) The standard curve of quantitative detection of RC0497 in spiked human samples. Limit of detection (LoD) was estimated as the analyte concentration with signal above the average of blank+3 SD (n = 5). Serum samples of two experimental models of SFRs analyzed by LFA “version 1”, were pooled (n = 8) and 25% diluted in LFA running buffer. LFA strips for the animal serum samples were analyzed on both LFA readers. TL/CL were obtained for guinea pig (C) and mouse (D) serum samples. In the box plots, horizontal lines on each box plot, from bottom to top beginning with the bottom whisker are: 10th percentile, 25th percentile, median, 75th percentile, and 90th percentile. Replicates (n = 7) are shown in closed symbols, average values in open symbols.

While preparing for further clinical studies, newly-received lots of the 200 nm europium FluoSpheres began to show unexpectedly dim fluorescent signals and failed our QC testing. This necessitated a change to the 200 nm europium particles from Bangs Laboratories. At the same time, a more POC-compatible, low-cost, time-resolved europium fluorescence LFA reader (Lumigenex, LTRIC-600) became available. We confirmed the performance of the RC0497 LFA version 2 (which differs from the original LFA only in the use of particles from Bangs Laboratories and the LTRIC-600 reader) with a commercially available recombinant RC0497 protein of high purity (Genescript, #LOC107446314; not available during initial LFA development) spiked in negative human serum and by re-testing the previously-used guinea pig and mouse serum samples (Fig 4). By using spiked human samples, we determined that the LoD of the RC0497-europium LFA was 0.64 ng/mL (Fig 4A and 4B and S2 and S3 Tables). The results of samples of experimental models of SFRs analyzed by LFA “version 2” were not significantly different from those of LFA “version 1” (Fig 4C and 4D), suggesting the robustness of the RC0497-europium LFA.

## Discussion

We developed an LFA to detect the diagnostic biomarker specific for acute SFRs, RC0497, in serum specimens and confirmed its robust performance with varied europium reporter particles and two point-of-care LFA readers. The LFA used a Test Line (TL) of anti-RC0497 rabbit polyclonal antibodies, a Control Line (CL) of anti-rabbit IgG, and europium chelate nanoparticles functionalized with anti-RC0497 polyclonal antibodies as reporters. We evaluated the RC0497 EuNP LFA using a panel of serum specimens from experimentally infected animals and spiked human serum samples.

Compared to the current laboratory tests for rickettsioses [1], the RC0497-EuNP LFA is a potentially rapid diagnostic tool specifically designed to detect a rickettsial antigen. Accurate detection of RC0497 in 13 infected guinea pig specimens and 8 infected mouse samples, and in none of 7 uninfected specimens, demonstrated that TL/CL ratios correlated with the presence of RC0497 and were associated with the acute infection in the experimental models of SFRs, suggesting that detection of RC0497 by EuNP LFA could potentially serve as a POC test for rickettsial diseases. Using the mouse model, we demonstrated that day 3 p.i. is the earliest time point at which RC0497 was detectable by LFA over the course of the infection. It is worth noting that mice began to show signs of illness by day 2 after infection, whereas guinea pigs exhibited fever starting on day 3 p.i.. All serum samples collected on days 4 to 5 p.i. from infected guinea pigs and on day 3 p.i. from infected mice were found to be positive when tested using the EuNP LFA (Figs 2 and 3). Therefore, the earliest time point for detecting RC0497 by LFA is estimated to be approximately on days 1~2 following the onset of clinical signs. The RC0497 LFA operates by evaluating the TL/CL ratio using a europium LFA reader, for minimized subjectivity and straightforward interpretation. Furthermore, one of the advantages of utilizing experimental models to evaluate LFA is their capability to determine the timely potential for improving infection outcomes by measuring bacterial load. Our findings indicate that doxycycline treatment on day 3 after illness onset significantly controlled the infection so that the RC0497 LFA is expected to offer prompt, actionable guidance in making therapeutic decisions.

While detection of RC0497 by LFA appears promising, this study is based on a limited number of experimentally infected animals. A comprehensive evaluation of the performance of the RC0497 LFA using a large cohort of patients’ specimens is warranted. Additionally, assessing the practical utility of the test in clinical settings by healthcare professionals will address the question on whether the RC0497 LFA is applicable for both mild and severe SFRs, and whether RC0497 could serve as a prognostic biomarker.

Nevertheless, this is the first demonstration of a rapid antigen-detection test prototype based on a lateral flow assay platform for diagnosing life-threatening SFRs in a timely manner. We demonstrated the presence of a rickettsial antigen in the circulation of experimentally infected animals that was detectable by a simple test not requiring sophisticated equipment. The RC0497 LFA may distinguish active infections, in contrast to serological tests used for the detection of previous exposure to potentially nonpathogenic rickettsial species so that it will facilitate both clinical diagnosis and epidemiological surveillance. Although future studies validating the assay with patient specimens are required, we have established the proof-of-concept that this LFA could be translated to a point-of-care diagnostic assay in the real world.

## Supporting information

S1 Table Ratios of TL/CL of the studied samples collected from experimental models of SFRs. (TIF)

S2 Table Ratios of TL/CL of human sera spiked with recombinant RC0497. (TIF)

S3 Table Calculated ratios of TL/CL and limit of detection in spiked human serum samples. (TIF)
