# Supplementary figures and images for: Development of a rapid antigen-based lateral flow assay for tick-borne spotted fever rickettsioses
Source: PLoS One. 2025 Jan 17;20(1):e0312819. doi: 10.1371/journal.pone.0312819 (PMC11741651; doi:10.1371/journal.pone.0312819)

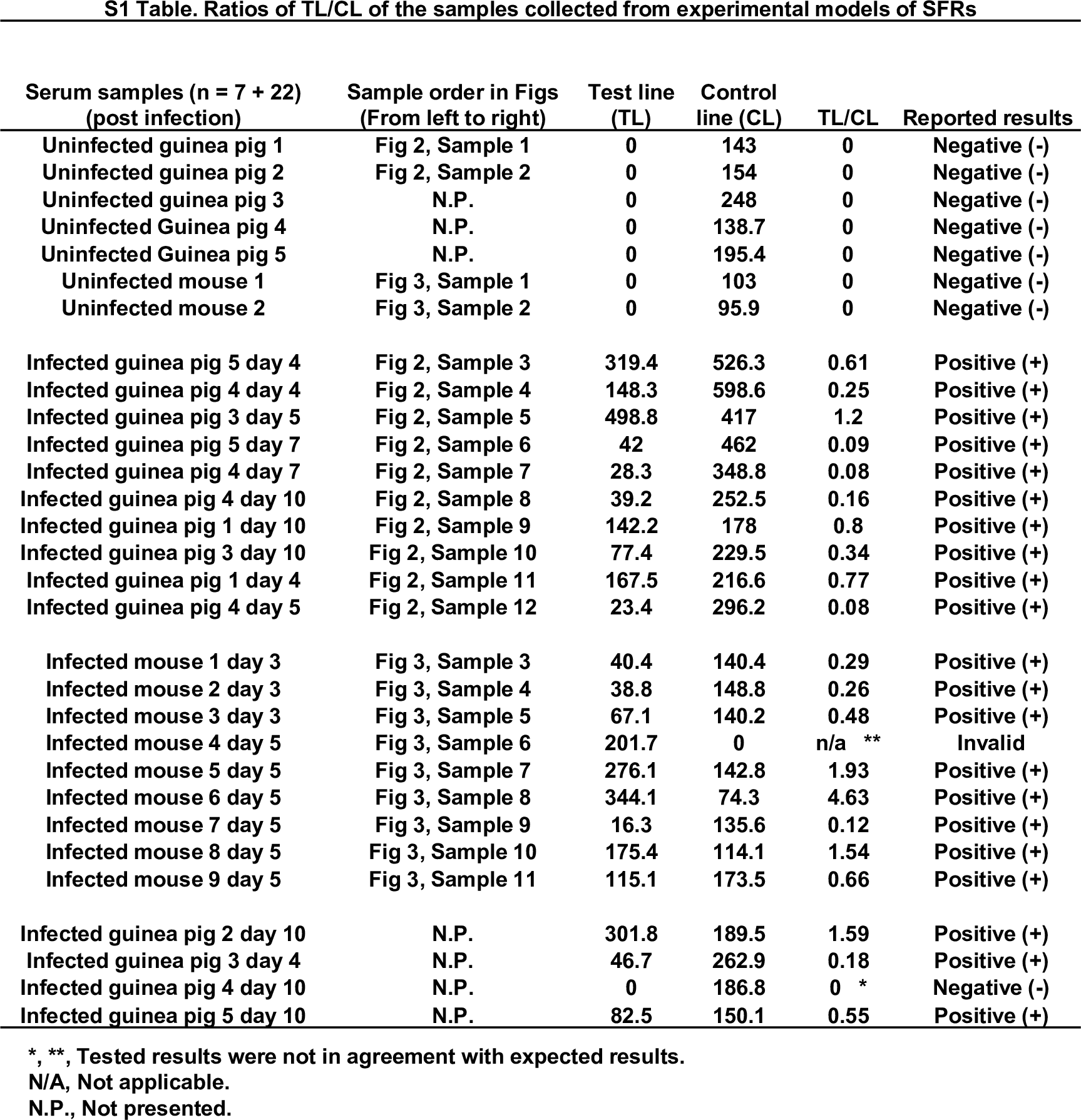

Supplement: S1 Table — (TIF) [file pone.0312819.s001.tif]

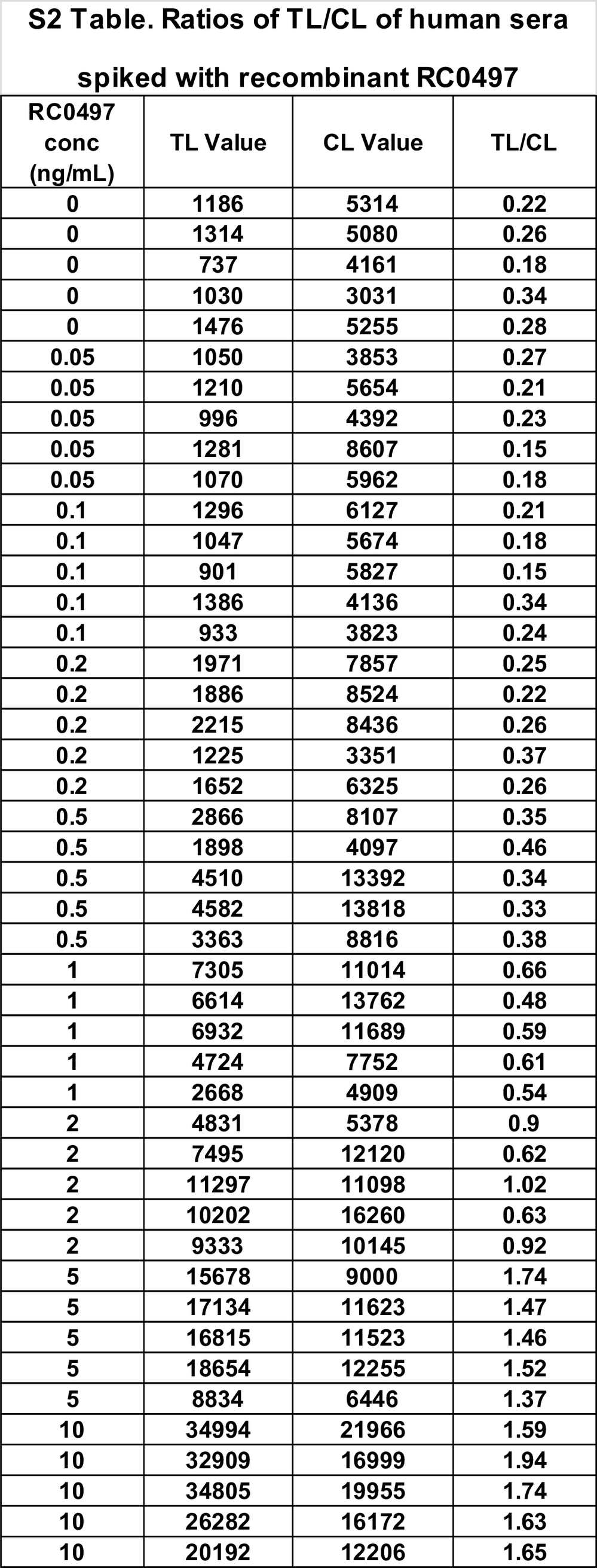

Supplement: S2 Table — (TIF) [file pone.0312819.s002.tif]

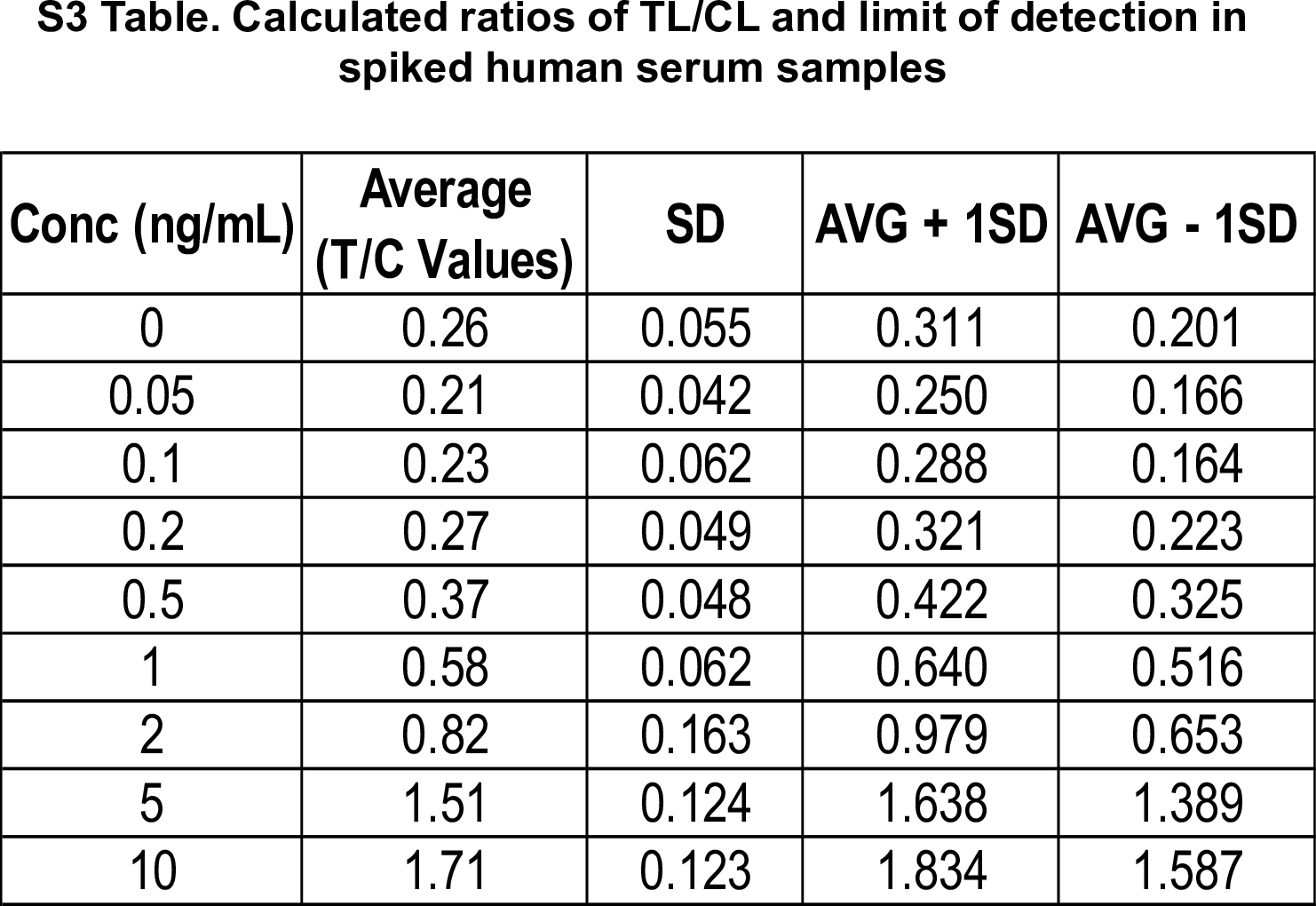

Supplement: S3 Table — (TIF) [file pone.0312819.s003.tif]
